# Proteomic Analysis of Rapeseed Root Response to Waterlogging Stress

**DOI:** 10.3390/plants7030071

**Published:** 2018-09-07

**Authors:** Jinsong Xu, Xing Qiao, Zhitao Tian, Xuekun Zhang, Xiling Zou, Yong Cheng, Guangyuan Lu, Liu Zeng, Guiping Fu, Xiaoyu Ding, Yan Lv

**Affiliations:** Key Laboratory of Biology and Genetic Improvement of Oil Crops, Ministry of Agriculture, Oil Crops Research Institute of the Chinese Academy of Agricultural Sciences, Wuhan 430062, China; xujinsong@caas.cn (J.X.); qiaoxing@caas.cn (X.Q.); tianzhi_tao@163.com (Z.T.); zhang.xk@139.com (X.Z.); zouxiling@gmail.com (X.Z.); 13808614864@139.com (Y.C.); luwiz@163.com (G.L.), zengliu0929@163.com (L.Z.); gpf709@126.com (G.F.); dingxiaoyu1991@163.com (X.D.)

**Keywords:** Keywords: rapeseed, iTRAQ, proteomics, waterlogging stress

## Abstract

The overall health of a plant is constantly affected by the changing and hostile environment. Due to climate change and the farming pattern of rice (*Oryza sativa*) and rapeseed (*Brassica napus* L.), stress from waterlogging poses a serious threat to productivity assurance and the yield of rapeseed in China’s Yangtze River basin. In order to improve our understanding of the complex mechanisms behind waterlogging stress and identify waterlogging-responsive proteins, we firstly conducted iTRAQ (isobaric tags for relative and absolute quantification)-based quantitative proteomic analysis of rapeseed roots under waterlogging treatments, for both a tolerant cultivar ZS9 and sensitive cultivar GH01. A total of 7736 proteins were identified by iTRAQ, of which several hundred showed different expression levels, including 233, 365, and 326 after waterlogging stress for 4H, 8H, and 12H in ZS9, respectively, and 143, 175, and 374 after waterlogging stress for 4H, 8H, and 12H in GH01, respectively. For proteins repeatedly identified at different time points, gene ontology (GO) cluster analysis suggested that the responsive proteins of the two cultivars were both enriched in the biological process of DNA-dependent transcription and the oxidation–reduction process, and response to various stress and hormone stimulus, while different distribution frequencies in the two cultivars was investigated. Moreover, overlap proteins with similar or opposite tendencies of fold change between ZS9 and GH01 were observed and clustered based on the different expression ratios, suggesting the two genotype cultivars exhibited diversiform molecular mechanisms or regulation pathways in their waterlogging stress response. The following qRT-PCR (quantitative real-time polymerase chain reaction) results verified the candidate proteins at transcription levels, which were prepared for further research. In conclusion, proteins detected in this study might perform different functions in waterlogging responses and would provide information conducive to better understanding adaptive mechanisms under environmental stresses.

## 1. Introduction

Rapeseed is an important oil crop, due to the high content of oil in the seed, and it accounts for one-third of edible oils around the world [1]. However, rapeseed is sensitive to various environmental stresses, and abiotic stress conditions, such as drought, salinity, flood, and cold, severely limit the growth and production of crop, resulting in agricultural economic loss and agricultural production risks [2,3,4,5]. Waterlogging, also known variously as flood, submergence, soil saturation, anoxia, and hypoxia, is a considerable agricultural problem around the world, and usually manifests as one of two types in the field: (1) “waterlogging” refers to the root and part of the shoot being submerged underwater; while (2) “complete submergence” refers to the entire plant being submerged underwater [6]. Since oxygen is necessary for respiration in roots, oxygen deficiency causes the main damage, as waterlogging always results in anoxic soils and hypoxia within roots, which limits root growth and consequently reduces shoot growth and yield [7,8]. In addition, waterlogging stress also raises the probability of a pathogen infection [9]. As a result, improvement to water stress tolerance has become an urgent priority for crop breeding programs [6,10].

Crops have evolved and developed various acclimation strategies at morphological, cellular and metabolic levels, such as the formation of adventitious roots and aerenchyma, increased soluble sugar content, enhancement of the glycolytic pathway, activation of antioxidant defense, and triggered innate immunity [6,11,12]. At the soil surface, under oxygen-deficient conditions, the formation of adventitious roots has been universally known as an important method of waterlogging stress adaption in different species [13]. Previous studies have implicated that when the oxygen supply is difficult to maintain, plants initiate organogenesis with adventitious roots emerging from stem nodes, thus improving gas diffusivity around the root [14,15]. In various plants species, the significance of ROS or hormonal regulation in adventitious roots has been reported extensively [15,16,17,18], and adventitious root formation has been successfully applied to be the root system architecture in genetic resource development or identification [19,20,21,22,23]. However, the process is largely unknown in rapeseed. To date, there have been many achievements in the discovery of waterlogging mechanisms. Ethylene is the primary signal for most adaptations under waterlogging stress condition. When rice becomes submerged, ethylene promotes root emergence from the nodes by inducing ROS formation and cell death in the epidermal cells [24]. Similarly, in *Solanum dulcamara*, ethylene co-opts the ABA and auxin signaling cascades to regulate root development under flooded soil condition [25]. In wheat, it has also been reported that ethylene could induce the expression of genes correlated with aerenchyma formation, glycolysis, and a fermentation pathway [7]. Gene functional analysis demonstrates that the VII ethylene response factors (ERFs) play a crucial role in the regulation of the waterlogging response. Submergence-1 (Sub1) is a major quantitative trait locus (QTL) affecting submergence tolerance in rice. This QTL in the tolerant line encodes *SUB1A*, which belongs to the group VII ERF in rice, limits the GA responsiveness promoted by ethylene. The QTL functions by using a quiescence strategy that economizing carbohydrate reserves, thus helping keep the plant alive in submergence conditions and regrow it after submergence [26,27,28]. Meanwhile, two other ERFs, *SNORKEL1* (SK1) and *SNORKEL2* (SK2), trigger the internode elongation via the promoting gibberellin pathway under deep water conditions [29]. Furthermore, five members of group VII ERF in *Arabidopsis*—HRE1, HRE2, RAP2.12, RAP2.2, and RAP2.3—have been proven to participate redundantly in the anaerobic response [30,31,32]. However, mechanisms in rapeseed still need to be established, and the gene or genes responsible for waterlogging tolerance have not been identified.

In recent years, research efforts based on the transcriptome technique have been applied to gain a preliminary understanding of the adaptive mechanisms adopted by plants in combating waterlogging stress. Studies have demonstrated that waterlogging stress in root leads to drastic expression changes of genes related to oxidation reduction, secondary metabolism, transcription regulation, and translation regulation [33,34]. In addition, waterlogged rapeseed leaves respond to hypoxia by regulating genes related to the scavenging of reactive oxygen, degradation (proteins, starch, and lipids), and premature senescence [35]. Regardless, transcriptome profiling has some limitations, because mRNA levels are not always correlated to corresponding proteins, mainly because of post-transcriptional regulation [36]. More recently, proteomic analyses were conducted on soybean and maize seedlings under different waterlogging conditions [37,38]. The iTRAQ combined with liquid chromatography–tandem mass spectrometry (LC-MS/MS) is a high throughput and stable strategy that explores dynamic changes in protein abundance with a highly accurate quantitation of different proteins, especially for lowly-expressed proteins [39,40,41]. Thus far, considerable work focusing on various stress traits has been carried out—for example, waterlogging stress on cucumber [42] and maize [38], salt stress on rapeseed [43,44], maize [45] and wheat [46], heat stress on *Arabidopsis* [47], and artificial aging in rapeseed [45]. However, to date, limited information is available about waterlogging-response proteins in rapeseed. This has limited our understanding of the molecular mechanism adopted by this important crop in response to waterlogging stress.

Waterlogging stress has significant effects on rapeseed (*Brassica napus* L.) at all stages of development, and our previous study has outlined the waterlogging tolerance coefficient (WTC) for evaluating waterlogging tolerance [34]. The transcriptome under waterlogging stress was then assayed in the tolerant variety ZS9 with 4432 differentially expressed genes identified [33]. In order to explore more responsive genes under waterlogging condition, the tolerant cultivar ZS9 plus a sensitive cultivar GH01 were used in this study, and iTRAQ-based quantitative proteomic analysis approach was firstly applied to rapeseed root at the germination stage. The main objective is to identify whether differentially expressed proteins associated with waterlogging stress depend on genetic background or not, which would be beneficial to resolving the molecular mechanism in responses to waterlogging stress. Thus, this analysis provides deeper insights into the effects of waterlogging stress on rapeseed root at the germination stage.

## 2. Results

### 2.1. Effects of Waterlogging Stress on Root Growth

**Figure 1:**
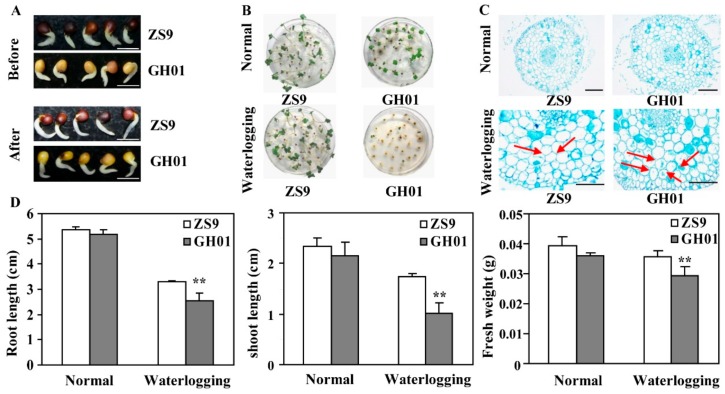
Effects of waterlogging stress on root growth. (**A**) Phenotypes of germinated seeds from ZS9 and GH01 before and after waterlogging stress for 12 h (bar = 0.5 cm); (**B**) Phenotypes of seedling emergence from ZS9 and GH01 under normal conditions and waterlogging stress conditions; (**C**) Transverse view of the ZS9 and GH01 radicles before and after waterlogging stress for 12 h. The sections were excised from root at 0.5 cm above the root tips, stained with toluidine blue, and photographed under stereoscopic microscope (scale bar = 0.25 mm); (**D**) Root length, shoot length, and fresh weights of ZS9 and GH01 under normal conditions and waterlogging stress conditions for three days (asterisks indicate statistically significant differences between ZS9 and GH01 at ** *p* < 0.01). Error bars present standard deviations based on three biological repetitions.

Based on the standard of the waterlogging stress condition, roots are the first to be affected by stress condition; thus, effects on roots were studied in detail. Rapeseed seeds of the tolerant cultivar ZS9 and sensitive cultivar GH01 were germinated for 36 h until their radicle grow out, then treated with or without waterlogging stress. Phenotypes show that the root growth of GH01 was repressed significantly compared with ZS9 after a 12-hour treatment (Figure 1A); in addition, under constant treatment for three days, GH01 had a lower seedling rate than ZS9 (Figure 1B). The cytological observation indicates that ZS9 and GH01 had a similar cell structure in the radicle, but different morphological changes within 12 h of waterlogging, as red arrows showed, with more parenchyma cells having withered in GH01 (Figure 1C). In addition, the root length, shoot length, and fresh weights of rapeseeds were measured under normal conditions and waterlogging stress conditions for three days (Figure 1D). The present results clearly demonstrate that the growth of the two cultivars was significantly suppressed by waterlogging stress. Moreover, GH01 showed a shorter root length and shoot length, and a lower fresh weight than ZS9. These results suggest that ZS9 has stronger adaptability than GH01 under waterlogging stress conditions.

### 2.2. Protein Identification and Quantification

**Figure 2:**
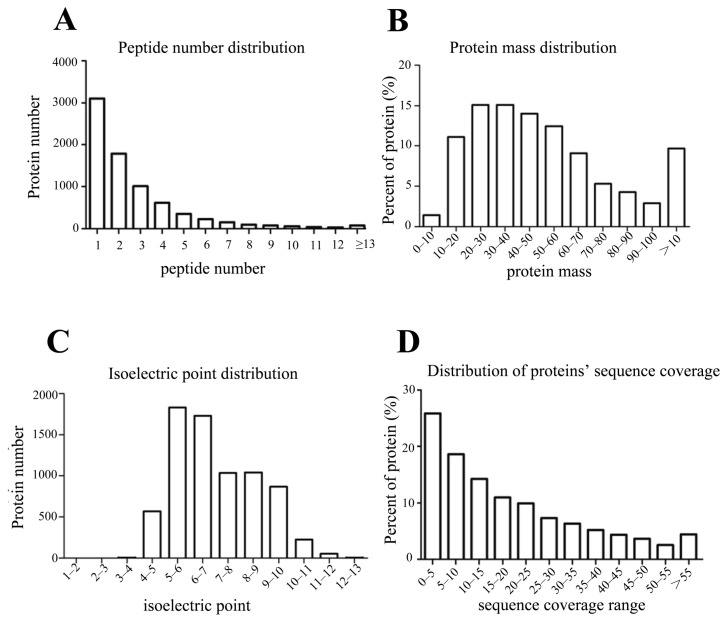
The distribution of peptide numbers, protein mass, isoelectric points, and sequence coverage of proteins identified, based on isobaric tags for relative and absolute quantification (iTRAQ) proteomics analysis.

The proteomes of ZS9 and GH01′s roots (ZS9-CK and GH01-CK, respectively) were collected before waterlogging stress and at 4 h (ZS9-4H, GH01-4H), 8 h (ZS9-8H, GH01-8H), and 12 h (ZS9-12H, GH01-12H) after waterlogging stress. The samples were then used for iTRAQ analysis. The peptides were searched against proteins derived from the *Brassica napus* L. genome database [48]. In total, 35,410 unique peptides corresponding to 7306 proteins were identified (Table S1). The number of peptides defining each protein is distributed in Figure 2A, and over 62% of the total (4529) proteins matched with at least two peptides. In addition, wide coverage was obtained on protein molecular weight (MW) distribution (Figure 2B), with the molecular weights of the identified proteins ranging from 3.3 to 570.6 kDa. Among them, 4774 weighed between 20 to 70 kDa, 914 between 0 to 20 kDa (low molecular weight proteins), 912 between 70 to 100 kDa, and 706 over 100 kDa (high molecular weight proteins), which show advantages in identifying proteins with low or high molecular weight compared to the traditional two-dimensional (2D)-gel technique. The isoelectric point distribution indicates that most of the identified proteins were between 5 to 10 isoelectric points (Figure 2C). Besides the above, the distribution of peptide sequence coverage is displayed in Figure 2D.

### 2.3. Differentially Expressed Proteins Response to Waterlogging Stress by iTRAQ

**Figure 3:**
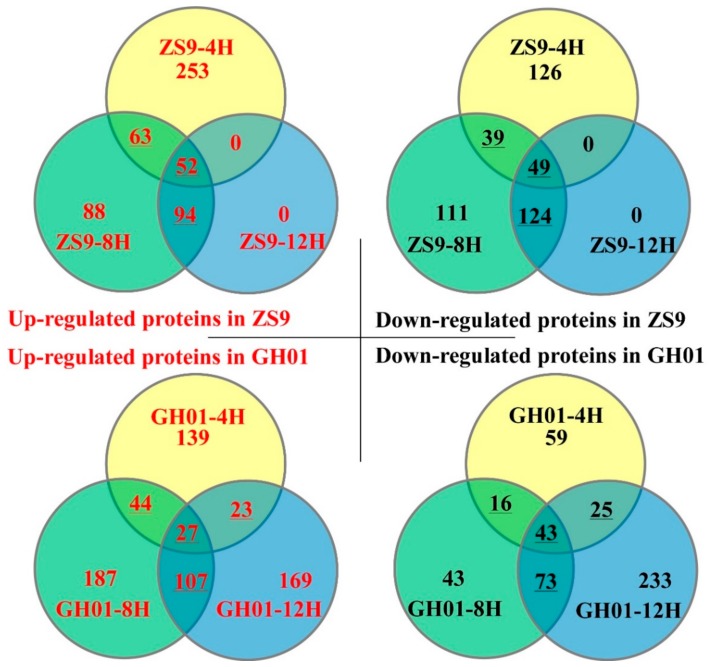
Venn diagram for the number of differentially expressed proteins in ZS9 and GH01 after undergoing waterlogging stress for 4 h, 8 h, and 12 h, the upward-regulated proteins or downward-regulated proteins are in a threshold ratio of >1.20 or <0.8.

The differences in protein abundance (fold change) for ZS9 and GH01 were based on the ratio of ZS9-4H/ZS9-CK, ZS9-8H/ZS9-CK, ZS9-12H/ZS9-CK, GH01-4H/GH01-CK, GH01-8H/GH01-CK, and GH01-12H/GH01-CK. Among the proteins that showed a significant change (*p* < 0.05 with a false discovery rate (FDR) of less than 5%) in abundance, we defined those with a ratio >1.20 as increased protein, and those with a ratio <0.8 as decreased proteins, which were chosen for further analysis. Among them, 368, 297, and 146 responsive proteins increased after undergoing waterlogging stress for 4 h, 8 h, and 12 h in ZS9, respectively, while 214, 323, and 173 responsive proteins decreased after undergoing waterlogging stress for 4 h, 8 h, and 12 h in ZS9, respectively. For GH01, there were 233, 365, and 326 increased proteins after undergoing waterlogging stress for 4 h, 8 h, and 12 h, respectively, and 143, 175, and 374 decreased proteins after undergoing waterlogging stress for 4 h, 8 h, and 12 h, respectively (Figure 3).

The Venn diagram shows a comparative analysis of the differentially expressed proteins mentioned above. As the dynamic detection for iTRAQ proteomics analysis contains three time points (4 h, 8 h, and 12 h), we focused on the differentially expressed proteins identified by at least two time points, which are underlined in Figure 3. These are more likely to be relevant to waterlogging stress and chosen for future study. For examples, 52 proteins continuously increased during stress in ZS9, while there were only 27 continuously increased proteins in GH01. Meanwhile, there were 49 and 43 continuously decreased proteins in ZS9 and GH01 during stress, respectively. These results indicate that variable proteins had dynamic and sustained changes over the course of waterlogging stress. The upward regulated proteins (ZS9-up) or downward regulated proteins (ZS9-down) identified based on the overlap in ZS9 are listed in Tables S2 and S3. The upward regulated proteins (GH01-up) or downward regulated proteins (GH01-down) identified based on the overlap in GH01 are listed in Tables S4 and S5.

### 2.4. Gene Onology Annotation of the Differentially Accumulated Proteins

**Figure 4:**
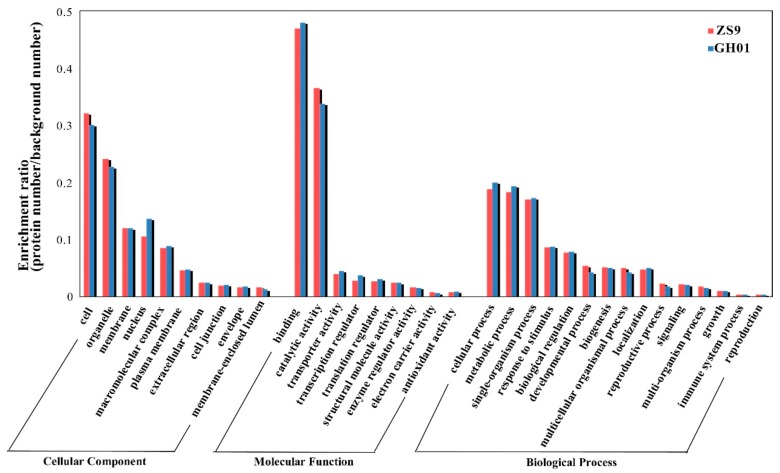
Histogram of significantly enriched proteins in gene ontology (GO) terms grouped with cellular component, molecular function, and biological processes between ZS9 and GH01. Left *y*-axis represents the ratio of proteins (protein numbers/background protein numbers) enriched in each GO term.

To further identify and annotate these proteins, we selected proteins belonging to ZS9-up, ZS9-down, GH01-up, and GH01-down groups (Figure 4) for gene ontology analysis by searching the NCBI protein database for homologous sequences using the BLASTp program, and 73–85% differentially accumulated proteins had GO annotation. The protein sequences were >40% identical with those of their homologs, suggesting that the proteins might have similar functions. These differentially accumulated proteins chosen from Figure 3 were classified into three groups (molecular function (GO-MF), cellular component (GO-CC), and biological process (GO-BP)) on the basis of GO enrichment analysis (Figure 4). Results show that enrichment of the main categories between ZS9 and GH01 are similar. The top two GO-CCs were “cell” with the percentage at about 32% and “organelle” with the percentage at about 35%, while there are higher ratios and a larger quantity of proteins in the classification of “nucleus” in GH01. This is consistent with the categories of “transcription regulator” in molecular functions, suggesting that the nuclear localized regulatory factors changing in GH01 might be negatively related to waterlogging stress. The top two GO-MFs were “binding activity”, with the percentage at about 47%, and “catalytic activity”, with the percentage at about 35%, respectively. For biological process, the enriched proteins mainly belong to the following categories: cellular (18.8–20.0%), metabolic process (18.4–19.4%), single-organism process (17.1–17.3%), response to stimulus (8.7%,) and biological regulation (7.8%).

Therefore, waterlogging stress in rapeseed affects sets of responsive proteins with different sub-cellular localizations and molecular functions, as well as different biological processes, indicating the diversity of response mechanisms, which need to be developed.

In order to further understand the specific biological process of differential accumulated proteins between ZS9-up, ZS9-down, GH01-up, and GH01-down groups, hierarchical cluster analysis was applied for the detailed biological process, based on the numbers of proteins belonging to the four groups (Figure S1). The clustering heat map shows that the enrichment of the four groups had both similarity and distinction. The GO-BPs with highest enrichment were the regulation of DNA-dependent transcription and oxidation-reduction processes, indicating that predominant responsive proteins after waterlogging stress were transcription factors, with proteins participating in oxidation–reduction process. We also observed that both ZS9 and GH01 contained proteins responsive to various stress and hormone stimuli, such as defense, water deprivation, wounding, oxidative, cold, and salt stress, as well as auxin, ethylene, auxin, jasmonic acid, and abscisic acid stimuli. This works together with some other reported mechanisms in stress responses, such as cell iron ion homeostasis, sucrose metabolic process, positive regulation of programmed cell death, lignin biosynthetic process, and peroxidase reaction, among others. However, proteins belonging to photorespiration, sodium ion transport, and ribofiavin metabolic processes were specific in sensitive cultivar GH01, while proteins annotated in defense response, hyperosmotic response, response to heat, response to cytokinin stimulus, and calcium ion transport were particular in tolerant cultivar ZS9. The results mentioned above suggest that cultivars with different level of waterlogging resistance exhibit similar and specific response mechanisms.

### 2.5. Pathway Analysis

**Figure 5:**
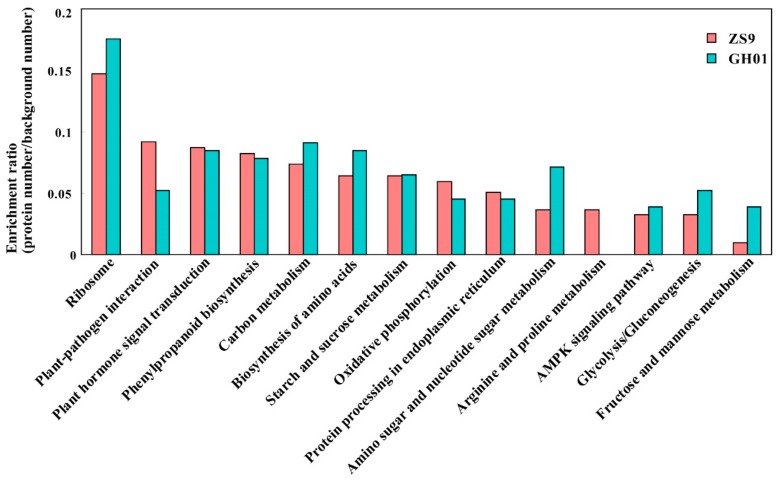
Histogram of significantly enriched proteins in the Kyoto Encyclopedia of Genes and Genomes (KEGG) pathways between ZS9 and GH01. Left *y*-axis represents the ratio of proteins (protein numbers/background protein numbers) enriched in each pathway.

To obtain an insight into the pathways in rapeseed roots during the course of waterlogging stress, we conducted a KEGG orthology based annotation system (KOBAS) analysis [49]. The significantly accumulated or decreased proteins in ZS9 and GH01 (ratio >1.2 or <0.8) (Tables S2–S5) identified in our iTRAQ data were subjected to KOBAS analysis, and then were mapped into different Kyoto Encyclopedia of Genes and Genomes (KEGG) pathways. A detailed view of the KEGG pathway is shown in Figure 5, and it was found to be enriched in ribosomes, plant-pathogen interaction, plant hormone signal transduction, carbon metabolism, biosynthesis of amino acids, oxidative phosphorylation, starch and sucrose metabolism, glycolysis/gluconeogenesis, and others (Figure 5). A significant difference was seen in the distribution frequency in the two cultivars. There were more proteins enriched in the carbon metabolism, especially fructose and mannose metabolism, for GH01. Moreover, it has been reported that in the initial flooding response, fructose functioned through the regulation of hexokinase and phosphofructokinase [50]. Meanwhile, for ZS9, special enrichment was observed in plant–pathogen interaction, as well as the arginine and proline metabolism, with these two pathways also reported in abiotic stress [51,52]. Therefore, we propose that the two cultivars developed different pathways to resist waterlogging stress, including metabolic-related functional proteins or regulators for fine-tuning plant stress response.

### 2.6. Differential Protein Overlapped with ZS9 and GH01

**Figure 6:**
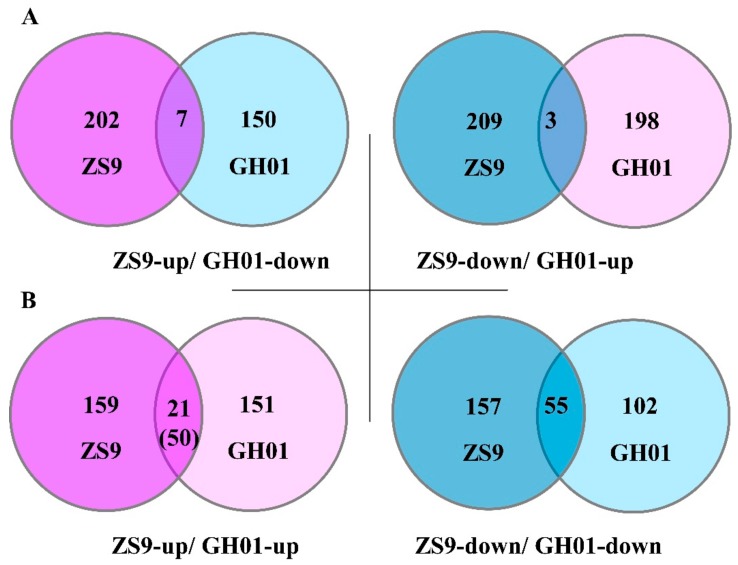
Venn diagram for the number of differentially expressed proteins with the same or opposite tendency between ZS9 and GH01—the threshold ratio is set as >1.20 or <0.8.

In order to find differentially expressed proteins based on the two genotype cultivars, we begin by comparing the proteins with the opposite tendency of fold change between tolerant cultivar ZS9 and sensitive cultivar GH01 (ZS9-up overlapped with GH01-down; ZS9-down overlapped with GH01-up) (Table S6). Seven proteins increased in ZS9 and decreased in GH01, suggesting they might be positively related to waterlogging stress, while three proteins decreased in ZS9 and increased in GH01, which might have a negative correlation with waterlogging stress (Figure 6A). Some of these had homologous proteins in *Arabidopsis*, which have been reported: BnaA09g29780D (GSBRNA2T00090709001) has 82% similarity in identity to AT1G24110, in which encoding peroxidase [53], and scavenging reactive oxygen species is one of the mechanisms of various abiotic stress responses. GO analysis also predicted this gene is related to peroxidase activity and oxidation–reduction process. Meanwhile, BnaC08g02330D (GSBRNA2T00052894001) is negatively associated to waterlogging stress, showing similarity in 92% identity to homolog in *Arabidopsis*, which responses to osmotic, salt, cold and drought stress [54]. It was also important for ethylene signaling [55], suggesting it might be a novel negative regulator in waterlogging stress response.

Noticeably, more proteins with the same tendency overlapped between ZS9 and GH01 (ZS9-up overlapped with GH01-up; ZS9-down overlapped with GH01-down) (Table S7), and their ratio was compared for fold changes between waterlogging-tolerant cultivar ZS9 and waterlogging-sensitive cultivar GH01. Nearly half of the proteins displayed differentially upward (21 in 50) or downward (13 in 55) regulated levels (Figure 6B). Of these proteins, BnaA09g07120D (GSBRNA2T00005761001) accumulated after waterlogging stress in ZS9 and GH01, showing 86% similarity in identity to TGA1 in *Arabidopsis*, which encodes a bZIP transcription factor and responds to ethylene stimulate [56]. Meanwhile, there might be other mechanisms besides the ethylene pathway. BnaC02g24210D (GSBRNA2T00012422001) decreased in the two cultivars, and the homologous protein in *Arabidopsis* was shown to respond to jasmonic acid (JA) and be produced during leaf senescence [57]. These proteins mentioned above suggest various mechanisms for waterlogging stress response, and we will continue to focus on these candidate proteins in future research.

**Figure 7:**
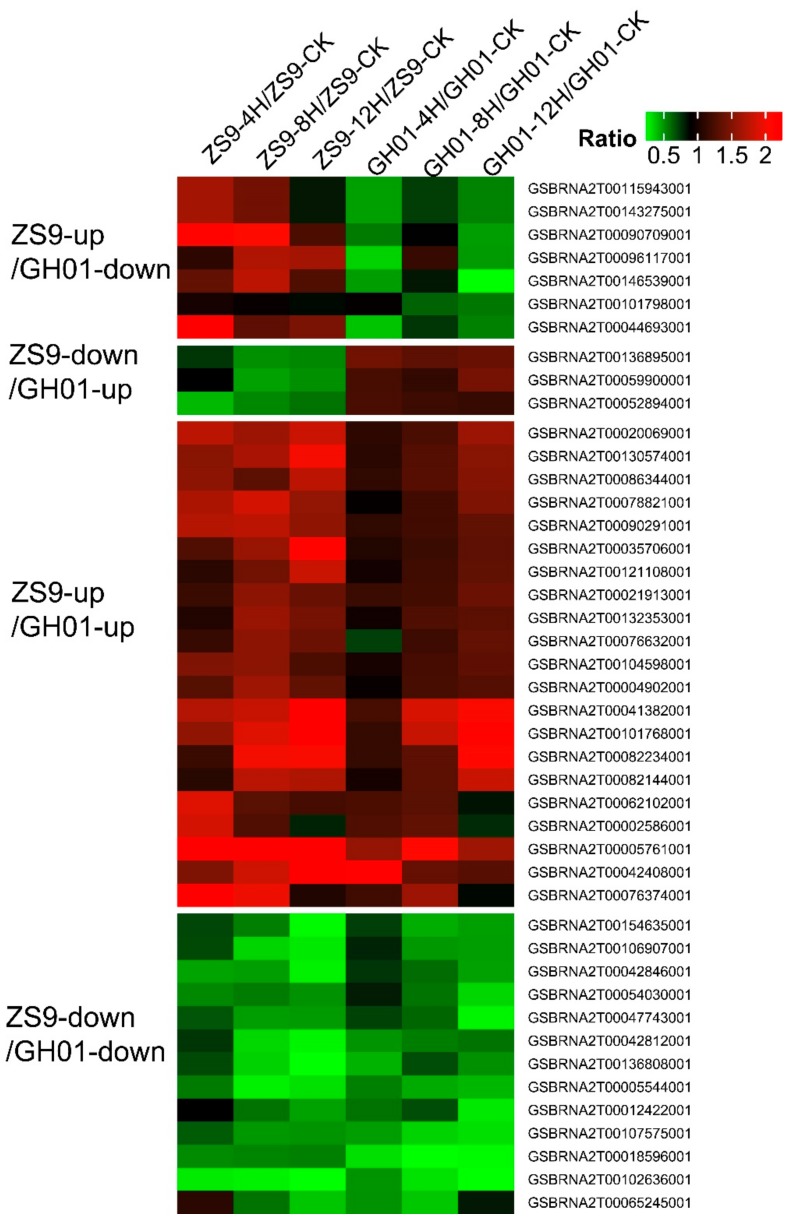
Heat map of the overlapped proteins between ZS9 and GH01 are clustered based on the differentially expressed levels. The original ratios (signal value after waterlogging stress/signal value before waterlogging stress) are color-coded, with green and red denoting decreased and increased expression, respectively.

**Figure 8:**
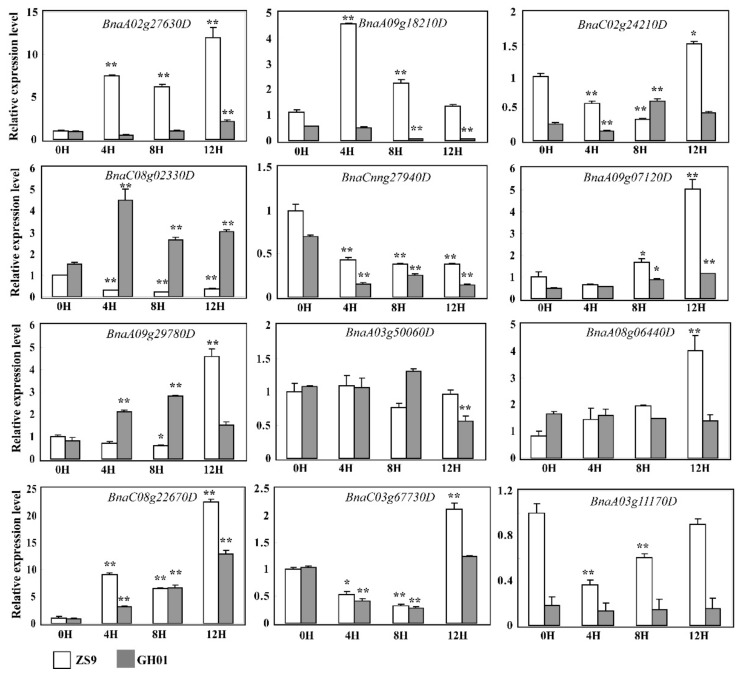
The relative expression level of candidate genes in ZS9 and GH01 germinated root before and after waterlogging stress by quantitative real-time polymerase chain reaction (qRT-PCR). Tissues used are indicated at the bottom. The values 0H, 4H, 8H, and 12H represent samples before waterlogging stress and after treatment for 4 h, 8 h, and 12 h, respectively. *BnaACT7* and *BnaUBC21* are amplified as internal reference. Asterisks indicate statistically significant differences between 0H and 4H, 8H, or 12H at ** *p* < 0.01 and * *p* < 0.05. Error bars indicate the standard errors based on three replicates.

For observing the aforementioned candidate proteins as a whole, a clustering method was applied to the threshold ratio of proteins overlapped by the ZS9-up/GH01-down, ZS9-down/GH01-up, ZS9-up/GH01-up, and ZS9-down/GH01-down groups, which corresponded to 7, 3, 21, and 13 proteins, respectively (Figure 7). The results show that in waterlogging-tolerant cultivar ZS9 and sensitive cultivar GH01, proteins functioning in different pathways changed in opposite or similar directions at different levels. In order to further study the functions of candidate genes in rapeseed, we are now preparing transgenic plants with FLAG tags or antibodies to verify the expression pattern at the protein level. Meanwhile, we performed qRT-PCR to confirm some of members at transcriptional level, which were generally consistent with iTRAQ data (Figure 8); the corresponding sequence name is listed in Table S8. The above results suggesting the diversiform molecular mechanism or regulation pathway in waterlogging stress response, which needs to be dissected.

## 3. Discussion

### 3.1. Contributions of Proteome Changes to Adapt to Waterlogging Stress

Waterlogging is a common environmental problem in rapeseed production, especially in the middle and lower reaches of the Yangtze River in China, where rice is usually the previous crop. Due to extensive flooding, waterlogging exposure leads to a decline in root development, nutrient transfer, seed setting rate, and rapeseed yield. As with the two cultivars ZS9 and GH01, our previous results showed that at germination stage, seedling stage, and maturity stage in the field, ZS9 displays a stronger ability to keep growth and recover under waterlogging stress conditions [34], in order to match our rapid screening method of waterlogging resistance that focuses on the germination stage [58], we kept on exploring response proteins at the germination stage, which are much less studied. In the present study, inhibition was observed for biomass accumulation in root and leaf tissue, and in seeding emergence (Figure 2), but the sensitive cultivar GH01 exhibited greater decline after undergoing waterlogging treatment.

Thus far, the application of the iTRAQ technique in exploring stress response proteins has been a heated issue. However, we noticed the higher number of waterlogging-responsive proteins in the GH01 cultivar, and a lower percentage of proteins changed in the early stage (4 or 8 h after waterlogging stress), while prolonging the treatment resulted in more proteins starting to response at 12 h. Nonetheless, response proteins at later stage could not rescue the plant from damage, thus GH01 reflected lower growth capability under stress condition (Figure 2). On the contrary, no special response proteins emerged at 12H after stress in the tolerant cultivar ZS9 (Figure 4), suggesting a quicker active proteome response in this tolerant cultivar. This phenomenon is possibly due to their different genetic backgrounds.

### 3.2. Waterlogging Stress Affects Proteins Involved in Ethylene Signaling

Many researches have shown that ethylene is a key mediator of waterlogging stress responses in plants [59], such as its important role in adventitious root or aerenchyma formation [18,60], and we did detect some ethylene response factors in ZS9-up and GH01-down. The accumulated protein BnaA02g27630D was homologous with EDF3, which promotes flower senescence or abscission and activates senescence-associated genes by ectopic expression in *Arabidopsis* [61], and premature senescence was known as a pathway for the root-waterlogged rapeseed resistant to hypoxia [35]. While BnaA09g18210D (GSBRNA2T00072354001) decreased in GH01-down, which displayed 63% similarity in identity to MACD1, that has been reported to positively regulate factors affecting programmed cell death [62]. Figure 8 showed these two members respond to waterlogging stress significantly with unique patterns, and the cluster analysis in Figure S1 also demonstrates that the rapeseed plant can cope with waterlogging through managing and regulating programmed cell death. Unexpectedly, few proteins out of the total were involved in ethylene signaling, indicating that the two genotype cultivars adopted various adaptive mechanisms to combat stress from waterlogging. On the other side, adventitious root would promote gas exchange, as well as uptake of water and oxygen, thus helping plants survive under low oxygen conditions; however, the adventitious root or aerenchyma formation always emerges many days after waterlogging stress [15,60]. According to previous standards for identification of resistance to waterlogging, we highlight the early stage within 12 h [58,63], so we presume that proteins related to adventitious root or aerenchyma formation are not yet starting to respond.

### 3.3. Waterlogging Stress Affects Proteins Involved in Protein Phosphorylation

Based on the GO cluster, the identified proteins include well-known classical pathways, such as ethylene response factors as well as pathways, including protein phosphorylation, the oxidation−reduction process, and regulation of transcription factors (Figure S1), which had good overlap with previous findings [33,35]. Despite these, other aspects, like protein phosphorylation or carbon metabolism, were poorly understood in rapeseed. Thus, it is certainly worthwhile to explore the responsible genes and resolve the response pathways.

As previously reported, protein post-translational modifications (PTMs) play a unifying and coordinating role in the plant. For instance, phosphorylation-mediated signaling mechanisms are associated with plant growth, development, and abiotic stress [64,65,66,67,68]. Studies also indicate that reversible protein phosphorylation constitutes a major event for perception and response to environmental and hormonal stimulates in plants [69,70]. Up until now, specific molecular mechanism based on protein phosphorylation in stress response has been a hot research topic [71,72]. Despite extensive studies, little is known about rapeseed. Yet, one study showed that calcium-dependent protein kinase (CDPK) members in rapeseed could interact with protein phosphatase 2C (PP2C) members, but the mechanisms need to be further studied [73]. In this study, members of proteins (GSBRNA2T00084051001, GSBRNA2T00109342001, GSBRNA2T00062102001, GSBRNA2T00062600001, etc.), including the serine/threonine protein kinase domain, were predicted to show protein phosphorylation activity (Tables S2–S5). In addition, GSBRNA2T00014963001 (BnaCnng27940D) decreasing in GH01 was predicted to encode a PP2C member, which has 78% similarity in identity to the PP2C5 in *Arabidopsis* that was reported to function in the early ABA signaling pathway [74,75]. The qRT-PCR results of our current work primarily confirmed the multifarious induction of these members under waterlogging stress conditions (Figure 8), whether the candidate proteins function through PTMs in rapeseed or not, we need to do more investigations, including phosphorylation assay, in further studies.

### 3.4. Waterlogging Stress Affects Proteins Involved in Metabolism

Previous papers indicate that plants respond to stress by modulating carbon metabolism in cell wall composition, including two main highlighted mechanisms: cell wall extensibility or polysaccharides and lignin synthesis, both of which help cells alleviate abiotic stress effects [76,77]. Xyloglucan endotransglucosylase/hydrolases (XTHs) are important cell wall enzymes that function in the modification of cell wall components by grafting xyloglucan chains to oligosaccharides or to other xyloglucan chains, and by hydrolysing xyloglucan chains [78,79]. Xyloglucan endotransglycosylase-1 (xet-1), a putative loosening enzyme in maize, was previously indicated in its involvement in response to root growth and oxygen deprivation [78,80]. In our study, xyloglucan endotransglucosylase hydrolase proteins (GSBRNA2T00081505001, GSBRNA2T00067403001, GSBRNA2T00131400001) (Tables S2–S5) were found to be differentially expressed under waterlogging stress conditions. Additionally, notably increased changes related to the biosynthesis of lignin were also observed in ZS9 and GH01 (GSBRNA2T00112348001, GSBRNA2T00116917001, GSBRNA2T00042846001, GSBRNA2T00121624001, etc.) (Figure S1). Lignin is one of the cell-wall components that has already been reported in defensive responses against clubroot in canola [81]; low water potential in maize [82]; flooding in cotton, wheat, and soybean [83,84,85]; and cold in plants [86]. Accordingly, it is clearly a necessity to undertake large-scale analysis to unravel the consequences of waterlogging stress on the cell wall in rapeseed.

## 4. Materials and Methods

### 4.1. Plant Material and Stress Treatments

According to our previous standard on waterlogging tolerance, the waterlogging-tolerant cultivar ZS9 and sensitive cultivar GH01 were chosen for this study. Seeds of the two rapeseed cultivars were sterilized with sterilized distilled water for 10 min. Then the seeds were germinated on wet filter paper in the dark for 36 h at 25 °C, until the root or radicle grow to 5mm long. Waterlogging stress was applied by adding distilled water until the germinated seeds are completely submerged. For iTRAQ sample preparation, 5 mm-long roots were collected at 0, 4, 8, and 12 h during the course of waterlogging treatment, then immediately frozen in liquid nitrogen before analysis. Three biological replications were mixed equally for each iTRAQ sample and qRT-PCR template.

After 12 h of waterlogging treatment, the germinated seeds were transferred to normal conditions for two days to observe seedling rate and measure root length, shoot length, and fresh weight. For normal conditions, rapeseeds were grown in a growth chamber illuminated with white fluorescent light (130 μmol m^−2^ s^−1^, 16 h light period/day) at 25 °C and 70% relative humidity.

### 4.2. Root Morphology and Optical Microscopic Observation

The experiment was performed as described previously [87]. Before and after 12 h of waterlogging treatment, the roots of germinated seeds from the two cultivars were put in 5% glutaraldehyde in a 50 mM phosphate buffer (pH 7.4), and then dehydrated in ethanol and embedded by white resin. The white resin section was stained with toluidine blue and then observed using optical microscope.

### 4.3. Protein Extraction

Protein extraction was conducted based on the trichloroacetic acid (TCA)/acetone method, with some modifications [88]. Roots were powdered in liquid nitrogen and suspended in 35 mL chilled (−20 °C) acetone containing 10% (*w*/*v*) TCA. The homogenate with incubation at −20 °C for 2 h was centrifuged at 7830 rpm for 30 min at 4 °C. The supernatant was carefully removed, then precipitation was washed three times with chilled acetone. Proteins were air-dried at room temperature and dissolved in 600 µL SDT buffer (4% SDS, 100 mM Tris-HCl, 1 mM DTT, pH 7.6), incubated in boiling water for 5 min under 100 Watts sonication, then boiled for 5 min and centrifuged at 13,400 rpm for 30 min at 4 °C. The supernatant was collected as a soluble protein fraction. The concentration of each extract was determined using a Bradford protein assay kit (Bio-Rad, Hercules, CA, USA) with bovine serum albumin (BSA) as a standard. The quality of each protein sample was tested using SDS-PAGE. Furthermore, tricine-sodium dodecyl sulfate polyacrylamide gel electrophoresis (Tricine-SDS-PAGE) was used to verify the quantitative results from the Bradford assay and determine the quality of the extract.

### 4.4. Protein Digestion and iTRAQ Labeling

**Figure 9:**
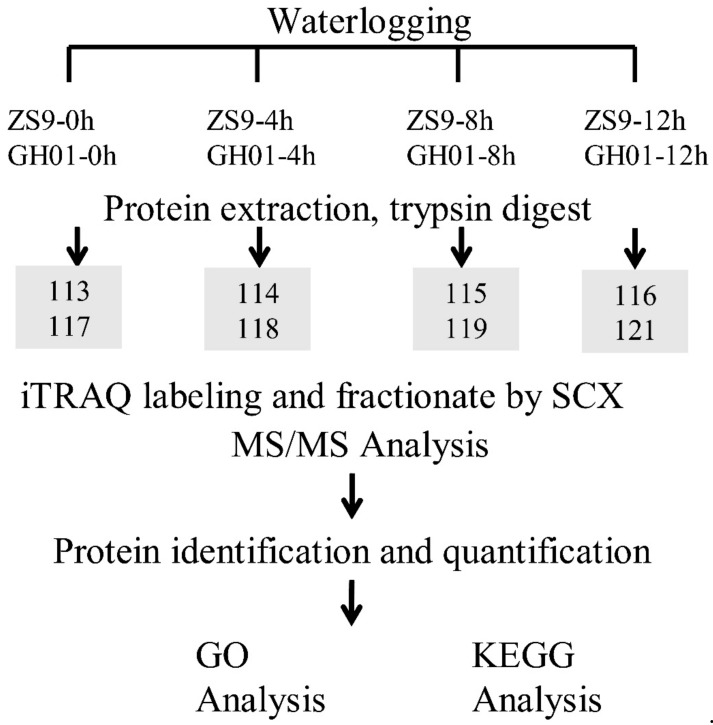
Experimental design of this proteomic study. After undergoing waterlogging stress for 0 h, 4 h, 8 h, and 12 h, the root of the germinated seed is sampled for protein extraction, trypsin digesting, and iTRAQ labeling. The iTRAQ-labeled samples are identified and quantified using the Easy-nLC Nanoflow HPLC system, and then identified proteins were taken for GO and KEGG analysis. Three biological replications are mixed equally for each iTRAQ sample.

The following experiments were carried out according to the technology roadmap in Figure 9. About 400 μg of mixed protein samples were added in 100 mM DTT, incubated in boiling water for 5 min, cooled to room temperature, diluted with 200 μL UA buffer (8 M urea, 150 mM Tris-HCl, pH 8.0), and subjected to 10 kDa ultrafiltration. The samples were centrifuged at 14,000× *g* for 15 min; 200 μL UA buffer were added and centrifuged for another 15 min. After adding 100 μL IAA buffer (0.05 M IAA), the samples were vibrated at 6000 rpm for 1 min, incubated for 30 min in darkness, and then centrifuged at 14,000× *g* for 10 min. After washing the filters three times with 100 μL UA buffer, 100 μL dissolution buffer was added and centrifuged for 10 min. This step was repeated twice, and then 5 μg trypsin (Promega) in 40 μL dissolution buffer was added to each filter; then the samples were vibrated at 6000 rpm for 1 min. The samples were incubated overnight at 37 °C. The resulting peptides were collected by centrifugation. The filters were rinsed with 40 μL dissolution buffer and centrifuged again. Finally, the peptide content was tested by spectral density with UV light at 280 nm [89].

About 80 μg peptides of each sample were labeled with iTRAQ reagents following the manufacturer’s instructions (Applied Biosystems, Thermo Fisher Scientific Corporation, Waltham, MA, USA), by using iTRAQ 8-plex kits (AB Sciex Inc., Framingham, MA, USA). After labeling, the samples were combined and lyophilized. The iTRAQ-labeled peptides were fractionated by strong cation exchange (SCX) chromatography in an AKTA Purifier 100 (GE Healthcare, Chicago, IL, USA) system equipped with a Polysulfethyl (PolyLC Inc., Columbia, MD, USA) column. The peptides were eluted at a flow rate of 1 mL/min. Buffer A consisted of 10 mM KH_2_PO_4_ and 25% *v*/*v* ACN, pH 3.0, and Buffer B consisted of 10 mM KH2PO4, 25% *v*/*v* ACN and 500 mM KCl, pH 3.0. The two buffers were filter-sterilized. The following gradient was applied to perform separation: 100% Buffer A for 25 min, 0–10% Buffer B for 7 min, 10–20% Buffer B for 10 min, 20–45% Buffer B for 5 min, 45–100% Buffer B for 5 min, 100% Buffer B for 8 min, and finally 100% Buffer A for 15 min. The elution process was monitored by measuring absorbance at 214 nm, and fractions were collected every 1 min. The collected fractions were finally combined into eight pools and desalted on C18 cartridges (Empore TM SPE Cartridges C18 (standard density), Sigma-Aldrich, St. Louis, MO, USA). Each fraction was concentrated via vacuum centrifugation and reconstituted in 40 µL of 0.1% *v*/*v* trifluoroacetic acid. All samples were stored at −80 °C until LC-MS/MS analysis.

### 4.5. Liquid Chromatography-Tandem Mass Spectrometry

The iTRAQ-labeled samples were analyzed using Easy-nLC nanoflow HPLC system connected to Q-Exactive mass spectrometer (Thermo Fisher Scientific, San Jose, CA, USA). A total of 5 μg of each sample was loaded onto Thermo Scientific EASY column (2 cm × 100 μm, 5 μm C18), using an auto-sampler at a flow rate of 0.3 mL/min. The sequential separation of peptides on a Thermo Scientific EASY column (100 mm × 75 μm, 3 μm C18) was accomplished using a segmented 1-h gradient from Solvent A (0.1% formic acid in water) to 35% Solvent B (84% ACN in 0.1% formic acid) for 50 min, followed by 35-100% Solvent B for 4 min, and then 100% Solvent B for 6 min. The column was re-equilibrated to its initial highly aqueous solvent composition before analysis. The mass spectrometer was operated in positive ion mode, and mass spectrometry (MS) spectra were acquired over a range of 300–1800 m/z. The resolution powers of the MS scan and MS/MS scan at 200 *m*/*z* for the Q-Exactive were set at 70,000 and 17,500, respectively. The top ten most intense signals in the acquired MS spectra were selected for analysis. The isolation window was 2 m/z, and ions were fragmented through higher energy collisional dissociation, with normalized collision energies of 30 eV. The maximum ion injection times were set at 10 ms for the survey scan and 60 ms for the scan, and the automatic gain control target values for both scan modes were set at 3.0 × 10^−6^. The dynamic exclusion duration was 25 s. The underfill ratio was defined as 0.1% on the Q-Exactive.

### 4.6. iTRAQ Analysis

The raw files were analyzed using the Proteome Discoverer 1.4 software (Thermo Fisher Scientific). A search for the fragmentation spectra was performed using the MASCOT search engine embedded in Proteome Discoverer against the *Brassica napus* genoscope database, which was downloaded from a *Brassica napus* L. filtered database (www.genoscope.cns.fr/brassicanapus). The following search parameters were used: mono-isotopic mass; trypsin as the cleavage enzyme; two missed cleavages; iTRAQ labeling and carbamidomethylation of cysteine as fixed modifications; peptide charges of 2+, 3+, and 4+; and the oxidation of methionine specified as variable modifications. The mass tolerance was set to 20 ppm for precursor ions and to 0.1 Da for fragment ions. The results were filtered based on a false discovery rate (FDR) of no more than 1% [90]. The protein identification was supported by at least two unique peptides.

The relative quantitative analysis of the proteins based on the ratios of iTRAQ reporter ions from all unique peptides representing each protein was performed using Proteome Discoverer (version 1.4). The relative peak intensities of the iTRAQ reporter ions released in each of the MS/MS spectra were used, and the reference (REF) sample was employed for calculating the iTRAQ ratios of all reporter ions. Thereafter, the final ratios obtained from the relative protein quantifications were normalized based on the median average protein quantification ratio. Protein ratios represent the median of the unique peptides of the protein. For quantitative changes, a 1.2-fold cutoff was set to determine upward-accumulated and downward-accumulated proteins, with a *p*-value < 0.05.

### 4.7. Bioinformatics Analysis

Functional analysis of proteins identified was conducted using GO annotation (http://www. geneontology.org/), and proteins were categorized according to their biological process, molecular function and cellular localization [91,92]. The differentially accumulated proteins were further assigned to the KEGG database (http://www.genome.jp/kegg/pathway.html) [93,94]. This study implemented FDR correction to control the overall Type I error rate of multiple testing using GeneTS (2.8.0) in the R (2.2.0) statistics software package. Pathways with FDR-corrected *p*-values < 0.05 were considered statistically significant.

### 4.8. RNA Extraction and qRT-PCR Analysis

The experiments were conducted as previously described [95]. Briefly, the RNA samples were collected from the root before and after waterlogging treatment at 4 h, 8 h, and 12 h at germination stage, which were exactly same with the samples for iTRAQ analysis. Then total RNA was extracted from the samples using TRIzol reagent (transgene) and then reverse transcribed to cDNA via Thermo kit protocol (Thermo Scientific RevertAid First Strand cDNA Synthesis Kit). The cDNA was then diluted 10-fold to perform qRT-PCR by using the PowerSYBR Green PCR Master Mix (Appliedbiosystems) on the StepOnePlus Real-Time PCR system, each reaction containing 0.4 μL specific primers, 2.0 μL cDNA, and 5.2 μL SYBR mixture. Three technical replicates were performed for each sample, and the program was set as following: 95 °C for 10 min; 42 cycles of 15 s at 95 °C, and 30 s at 60 °C. The melt curve, which aims to estimate the specificity of primers, was set from 65 °C to 95 °C with temperature increments of 0.5 °C for 5 s. The *BnaACT7* and *BnaUBC21* were used to standardize the RNA sample according to previous study [96,97,98]. The gene-specific primers used for qRT-PCR were designed on NCBI primer-blast and examined for specificity by BLASTn search in NCBI database, with all primers targeted mainly at the 3′ UTR; the amplification product size was between 70–250 bp. All primers related to this experiment are listed in Table S8. The statistically significance was determined through a *t*-test with ** *p* ≤ 0.01 and * *p* ≤ 0.05.

## 5. Conclusions

Our results show that iTRAQ is a powerful technique to perform quantitative proteome analysis of rapeseed roots under waterlogging stress. A large number of differentially expressed proteins responding to waterlogging stress were identified, and functional categorization of GO and KEGG analysis were applied, showing that the differentially changed proteins were enriched in the oxidation–reduction process, signal transduction, carbohydrate metabolism, and other processes. Quite a number of proteins constantly accumulated or decreased after undergoing waterlogging stress, with a quicker active proteome response in the tolerant cultivar ZS9. Cluster analysis was also applied to the differentially expressed proteins overlapped with the two cultivars, displaying that both similarity and distinction exist in waterlogging response mechanisms in the two genotypes. Taken together, our results show comprehensive proteome coverage of rapeseed roots in response to waterlogging treatments, and provide new insight into the molecular basis of waterlogging-stress response in rapeseed.

## Supplementary Materials

The following are available online at http://www.mdpi.com/2223-7747/7/3/71/s1. Figure S1. Heat map of differentially accumulated proteins clustered according to GO-BP terms based on the numbers. Table S1. All proteins identified through iTRAQ analysis. Table S2. The up-regulated proteins (ZS9-up) identified based on the overlap in ZS9. Table S3. The down-regulated proteins (ZS9-down) identified based on the overlap in ZS9. Table S4. The u- regulated proteins (GH01-up) identified based on the overlap in GH01. Table S5. The down-regulated proteins (GH01-down) identified based on the overlap in GH01. Table S6. Proteins with the opposite tendency of fold change between ZS9 and GH01. Table S7. Proteins with the same tendency of fold change between ZS9 and GH01. Table S8. Primers of candidate genes used for qRT-PCR.

## Abbreviations

CDPK	calcium-dependent protein kinase
ERFs	ethylene response factors
GO	Gene Ontology
iTRAQ	isobaric tags for relative and absolute quantitation
KEGG	Kyoto Encyclopedia of Genes and Genomes
LC-MS/MS	Liquid chromatography-tandem mass spectrometry
MAPK	mitogen-activated protein kinase
MS	mass spectrometry
PTMs	post-translational modifications
QTL	quantitative trait locus
qRT-PCR	quantitative real time polymerase chain reaction
TCA	tricarboxylic acid cycle
XTHs	xyloglucan endotransglucosylase/hydrolases
