# Supplementary material for: Proteomic Analysis of Rapeseed Root Response to Waterlogging Stress
Source: Plants (Basel). 2018 Sep 7;7(3):71. doi: 10.3390/plants7030071 (PMC6160990; doi:10.3390/plants7030071)
Supplement: Supplementary file 1 [file plants-07-00071-s001.zip › Supplementary Materials/Figure S1.doc]

**Figure S1**. Heat map of differentially accumulated proteins clustered according to GO-BP (gene ontology**-biological process**) terms based on the numbers.
